# Anti-Inflammatory Effects of *Hyptis albida* Chloroform Extract on Lipopolysaccharide-Stimulated Peritoneal Macrophages

**DOI:** 10.1155/2013/713060

**Published:** 2013-07-22

**Authors:** Elizabeth Sánchez Miranda, Julia Pérez Ramos, Cristina Fresán Orozco, Miguel Angel Zavala Sánchez, Salud Pérez Gutiérrez

**Affiliations:** Departamento Sistemas Biológicos, Universidad Autónoma Metropolitana-Xochimilco, Calzada del Hueso 1100, Colonia Villa Quietud, 04960 Mexico, DF, Mexico

## Abstract

We examined the effects of a chloroform extract of *Hyptis albida* (CHA) on inflammatory responses in mouse lipopolysaccharide (LPS) induced peritoneal macrophages. Our findings indicate that CHA inhibits LPS-induced production of tumor necrosis factor (TNF-**α**) and interleukin-6 (IL-6). During the process, levels of cyclooxygenase-2 (COX-2), nitric oxide synthase (iNOS), and nitric oxide (NO) increased in the mouse peritoneal macrophages; however, the extract suppressed them significantly. These results provide novel insights into the anti-inflammatory actions of CHA and support its potential use in the treatment of inflammatory diseases.

## 1. Introduction

Inflammation is an immediate response to many injuries produced by pathogens, noxious stimuli such as chemicals, or physical injury. Inflammation involves the activation and recruitment of phagocytes (macrophages, neutrophils), NK cells, the complement system, and the secretion of cytokines such as IL-1*β*, IL-6, and TNF-*α* by activated cells that are essential for the host defense system. Inflammatory disorders are treated using conventional anti-inflammatory drugs such as steroidal anti-inflammatory drugs and nonsteroidal anti-inflammatory drugs (NSAIDs) [1]. However, their prolonged use may produce adverse effects [2]. Thus, it is important to develop new anti-inflammatory agents with fewer adverse effects. Natural products can be a source of active metabolites that can serve as an alternate approach to anti-inflammatory drugs [3]. 

The genus *Hyptis* consists of approximately 400 species distributed from Southern United States to Argentina [4]. Plants in this genus have great economical and ethnopharmacological importance [5]. They have been used in folk medicine for the treatment of various disorders such as gastrointestinal disorders, skin infections, nasal congestion, fever, cramps, inflammation, and pain [5–8]. The genus *Hyptis* has many species that are important in Mexican folk medicine. In particular, *Hyptis albida* is commonly used in remedies for the treatment of gastrointestinal disturbances, skin infections, rheumatism, cramps, and muscular pains [9, 10]. Three triterpene lactones and five flavonoids have been isolated from an acetone extract [11] and the anti-inflammatory activity of a chloroform extract was reported by Pérez et al. [12]. The present investigation was carried out to assess the anti-inflammatory activity of a chloroform extract using murine macrophages stimulated with LPS.

## 2. Materials and Methods

### 2.1. Plant Material

Aerial parts of *H. albida* were collected in Guadalcazar, San Luis Potosi state, México. The plant was identified by taxonomist José García Pérez. A voucher specimen (SPLM 20419) was deposited in the Isidro Palacios Herbarium of the Universidad Autónoma de San Luis Potosi.

### 2.2. Preparation of the Extract

The shade-dried aerial parts were reduced to a powder, and 100 g of the powder sample was refluxed for 4 h with 400 mL chloroform. The extract was filtered, and the solvent was removed under reduced pressure (yield 5.3%). The extract showed positive results on Liebermann-Burchard, Tortelli-Jaffe, and Tschugaeff tests for terpenes and positive results on boric acid and citric acid for flavonoids [13].

### 2.3. Cell Culture

Macrophages were obtained from the peritoneal cavity of male BALB/c mice. Each mouse was injected with 1.5 mL of 4% thioglycollate in the peritoneal cavity. After 72 h a peritoneal lavage was performed with 10 mL cold 1x PBS buffer. The injected buffer was recovered and centrifuged to isolate cells. Cells were quantified using a Neubauer chamber and were cultured in plates for 24 h. Nonadherent cells were removed, and adherent cells were cultured in fresh medium. Peritoneal macrophages were maintained with RPMI supplemented with inactivated fetal bovine serum (FBS) at 10% and antibiotics, penicillin (100 units/mL) and streptomycin (100 *μ*g/mL), in an atmosphere of 5% CO_2_ at 37°C.

### 2.4. Cell Viability Using a Crystal Violet Exclusion Assay

Cells (1 × 10^6^ cells/well) were cultured in a 12-well plate for 24 h at 37°C in 5% CO_2_. After the medium was removed and replaced with fresh medium, CHA was added and incubated for 24 h. Viable cells were assessed with 0.4% crystal violet staining solution. Briefly, 200 *μ*L crystal violet was added to each well, and the cells were incubated for 30 min at room temperature until the crystal violet solution was changed to acid (33%). The solution was removed, and the absorbance was measured at 540 nm in a microplate reader [14].

### 2.5. Determination of Nitric Oxide Production

Peritoneal macrophages were cultured at a density of 1 × 10^6^ cells/well and incubated overnight. The macrophages were then pretreated with CHA at different noncytotoxic concentrations and incubated for 2 h. Then, LPS (1 *μ*g/mL) was added followed by incubation for a further 24 h. The cell supernatant was collected for nitrite analysis, and the pellet cell was used for the PCR assay. Nitrite production, an indicator of NO synthesis, was measured in the supernatant of cultured macrophages using the Griess reaction [15]. Briefly, equal volumes (100 *μ*L) of treated culture supernatant and Griess reagent (1% sulphanilamide, 0.1% NEDD, and 5% orthophosphoric acid) were mixed and incubated at room temperature for 5 min, and the absorbance was measured at 540 nm in a microplate reader. The amount of nitrite in the sample was determined using a sodium nitrite standard curve. 

### 2.6. RNA Isolation and RT-PCR Analysis

Total RNA from LPS-treated macrophages was extracted with the TRIzol reagent, according to the manufacturers protocol. RNA was stored at 70°C until used. Reverse transcription of RNA (1 *μ*g) was carried out with M-MuLV reverse transcriptase (Promega, WI, USA) and oligo-(dT)_18_ primers. PCR was performed in a reaction mixture containing the resulting cDNA, dNTP mixture (Promega), 10 pmol of target gene-specific primers, and 0.25 units of Taq DNA polymerase (Promega). Primers were designed using the Primer-BLAST software (http://www.ncbi.nlm.nih.gov/tools/primer-blast) (Table 1). PCR products were electrophoresed on 2% agarose gels and stained with Gel-Red.

### 2.7. Measurement of Proinflammatory Cytokines (TNF-*α* and IL-6) Production

Peritoneal macrophages were cultured at a density of 2 × 10^6^ cells/well and incubated overnight. Cell cultures were pretreated with different concentrations of CHA for 2 h thereafter; LPS (1 *μ*g/mL) was then added followed by incubation for a further 24 h. The inhibitory effect of CHA on the production of proinflammatory cytokines (IL-6 and TNF-*α*) was determined in the supernatants. Quantities of cytokines were measured using a mouse ELISA kit (eBioscience).

### 2.8. Statistical Analysis

All values are expressed as the mean ± SEM. Differences between mean values of normally distributed data were assessed with one-way ANOVA (Newman Keuls *t*-test). Statistical significance was accepted at *P* < 0.05.

## 3. Results

### 3.1. Effects of *H. albida* Extract on Cell Viability

**Figure 1:**
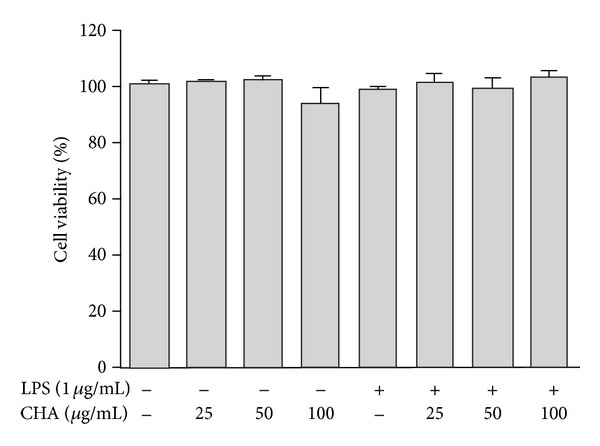
Effect of *H. albida* on the viability of peritoneal macrophages. The cells were treated with CHA in the absence or presence of LPS (1 *μ*g/mL) for 24 h. The values represent the mean ± SEM of three independent experiments.

The potential cytotoxicity of CHA was evaluated using the crystal violet assay after incubating cells for 18 h in the absence or presence of LPS. The results showed that cell viabilities were not affected by the extract at the indicated concentrations of 25, 50, and 100 *μ*g/mL (Figure 1). CHA did not show any cellular toxicity against peritoneal macrophages at these concentrations, which were then used in the experiments.

### 3.2. Inhibitory Effects of *H. albida *Extract on NO Production in Peritoneal Macrophages

**Figure 2:**
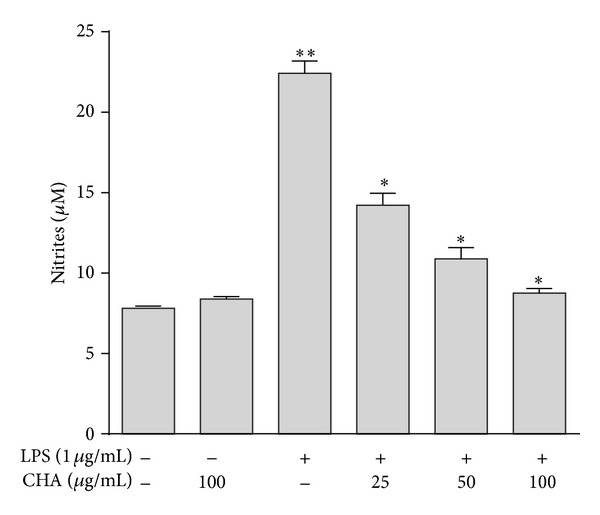
Effects of CHA at concentrations 25, 50, and 100 *μ*g/mL on production of NO in LPS-stimulated peritoneal macrophages. The values are the mean ± SEM of three independent experiments. **P < 0.05 LPS versus basal and group extract, *P < 0.05 versus LPS group.

Murine macrophages under basal conditions in culture media during incubation for 24 h produced 7.8 ± 0.12 *μ*M NO. CHA did not significantly affect the basal level of NO (8.4 ± 0.2 *μ*M). When cells were exposed to 1 *μ*g/mL LPS for 24 h, the nitrite concentration increased markedly, to 22.42 ± 1.41 *μ*M. CHA inhibited the production of NO after LPS stimulation in a dose-dependent manner by 36.62, 51.42, and 61.0% at 25, 50, and 100 *μ*g/mL, respectively, (Figure 2). No significant difference in NO level was found between resting cells and the cells treated with 100 *μ*g/mL of CHA.

### 3.3. Inhibitory Effects of *H. albida* Extract on iNOS and COX-2 mRNA Expression in LPS-Stimulated Peritoneal Macrophages

**Figure 3:**
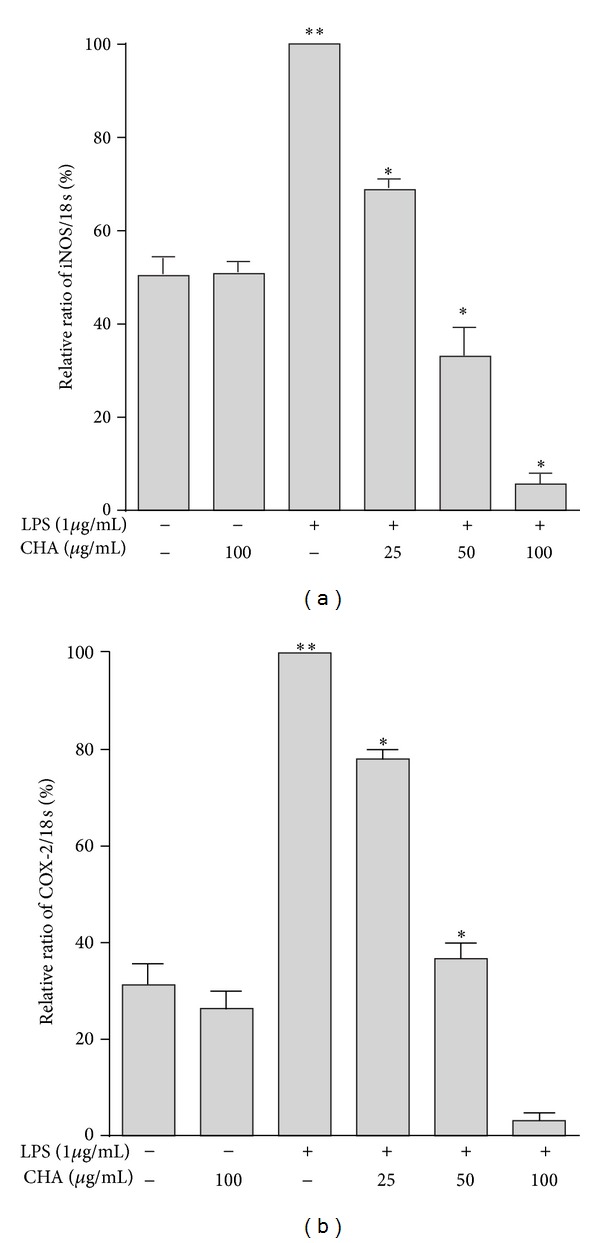
Effect of CHA on LPS-induced mRNA expression of iNOS (a) and COX-2 (b) in peritoneal macrophages. The mean values ± SEM for three independent experiments are shown. **P < 0.05 versus basal and group extract, *P < 0.05 versus LPS group.

COX-2 and iNOS are important enzymes in inflammation. To understand whether CHA can inhibit LPS-induced mRNA expression of these enzymes, a semiquantitative RT-PCR was performed. The expression of iNOS and COX-2 mRNA increased markedly upon LPS stimulation for 24 h, and CHA inhibited their expression in a concentration-dependent manner. iNOS was inhibited 31.22, 67.0, and 94.37% at 25, 50 and 100 *μ*g/mL, respectively (Figure 3(a)), and COX-2 by, 22.0, 63.3, and 96.97%, respectively, at the same concentrations (Figure 3(b)). These results suggest that suppression of iNOS mRNA was responsible for the inhibitory effect of CHA on LPS-stimulated NO production.

### 3.4. *H. albida* Extract Inhibits LPS-Induced Production of TNF-*α* and IL-6 in Peritoneal Macrophages

**Figure 4:**
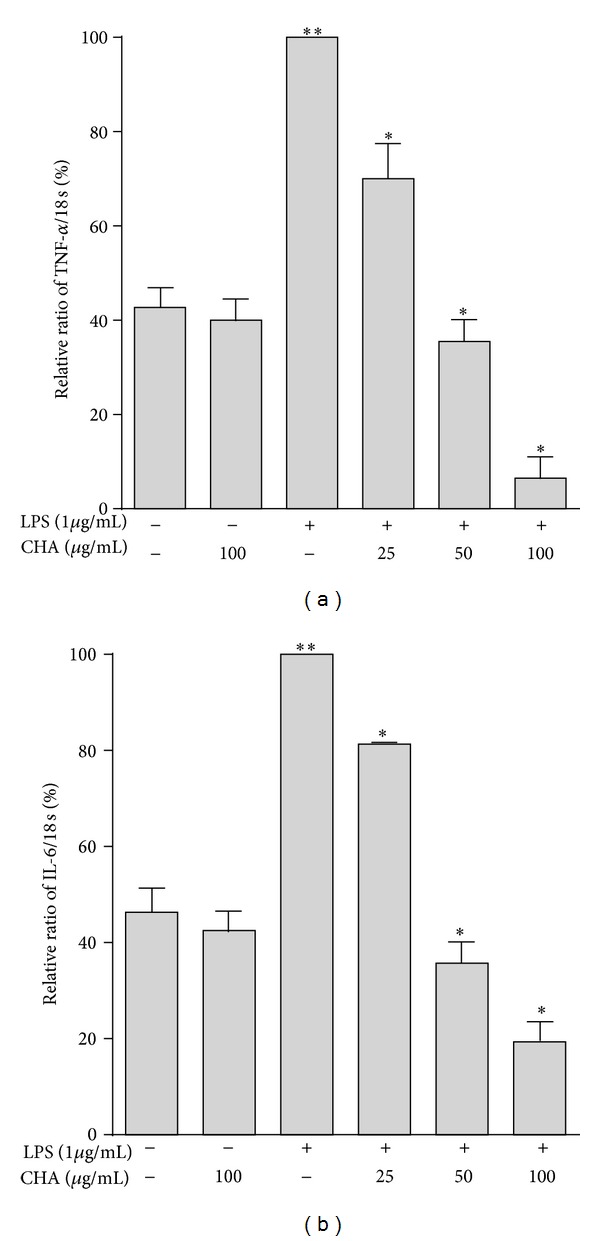
Effects of the extract of CHA on the (a) TNF-*α* and (b) IL-6 production in the peritoneal macrophages. Concentration in the supernatants was determined by ELISA. The results are the mean values  ± SEM for three independent experiments. **P < 0.05 versus basal and group extract, *P < 0.05 versus LPS group.

We assessed the effects of CHA on the production of the proinflammatory cytokines, TNF-*α*, and IL-6, in LPS-exposed cells. Secretion of these cytokines was measured in the culture media of cells stimulated with 1 *μ*g/mL LPS, alone or in combination with 25, 50, or 100 *μ*g/mL CHA. Cytokine levels were measured by ELISA. Treatment of the cells with LPS resulted in significant increases in cytokine production relative to the control group. However, macrophages pretreated with CHA showed significantly reduced TNF-*α*, and IL-6 production (Figure 4). In particular, TNF-*α* was inhibited by CHA in LPS-stimulated macrophages (74.05%) at 100 *μ*g/mL (Figure 4(a)). Moreover, IL-6 production was inhibited 15.0% at the same concentrations (Figure 4(b)). No significant difference was observed between resting cells and cells treated with 100 *μ*g/mL CHA.

### 3.5. Effects of CHA on mRNA Expression of TNF-*α* and IL-6 in LPS-Stimulated Macrophages

**Figure 5:**
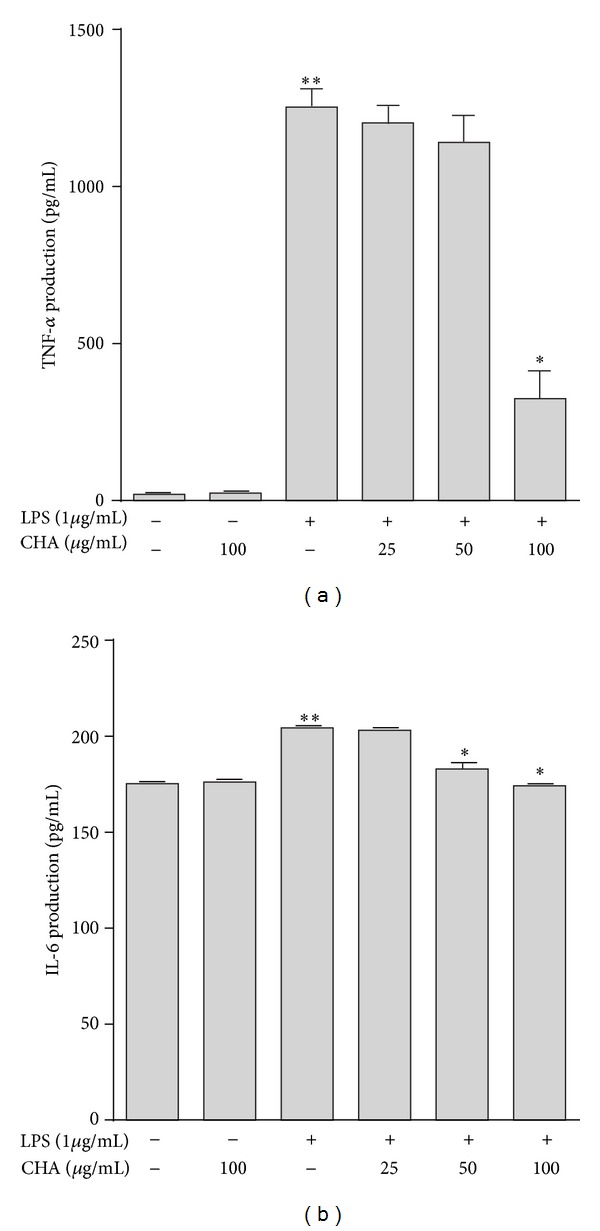
Effect of CHA on LPS-induced mRNA expression of TNF-*α* (a) and IL-6 (b) in peritoneal macrophages. The results are mean values ± SEM for three independent experiments. **P < 0.05 versus basal and group extract, *P < 0.05 versus LPS group.

RT-PCR was performed to determine whether CHA reduced the expression of these cytokines at the mRNA levels. The levels of TNF-*α* and IL-6 mRNA were upregulated markedly in response to LPS treatment (Figure 5). CHA inhibited the expression of both messengers significantly in a concentration-dependent manner. TNF-*α* was inhibited by 30.0, 64.5, and 93.53% at 25, 50, and 100 *μ*g/mL, respectively (Figure 5(a)), and IL-6 mRNA expression was suppressed by 18.67, 64.25, and 80.67% at the same concentrations (Figure 5(b)).

## 4. Discussion

Natural products play a significant role in drug discovery and development. The search for natural products with anti-inflammatory activity has increased markedly in recent years. *H. albida* is member of the Lamiaceae family, is restricted to the Pacific drainage of Mexico, and ranges from southwestern Sonora to central Guerrero. It is the medicinal plant used traditionally to treat various types of diseases [16]. 

Inflammation is a normal physiological and immune response to tissue injury and occurs when the human body attempts to counteract potentially injurious agents, such as invading bacteria, viruses, and other pathogens [17]. Macrophages play an important role in triggering inflammation during pathological conditions by overproducing inflammatory mediators, through the upregulation of inducible genes that contribute to inflammatory responses [18, 19]. LPS is an endotoxin, an integral outer membrane component of gram-negative bacteria. It induces the production of proinflammatory cytokines, NO, and prostaglandins (PGs) in macrophages [20]. Thus, therapeutic agents that inhibit the biosynthesis of these mediators may be useful for relieving proinflammatory conditions. 

Proinflammatory mediators, such as NO and PGE_2_, are generated via iNOS and COX-2, respectively [21]. NO is a signaling molecule and has been shown to have multiple physiological effects on various organ systems. Some of the most prominent physiological actions of NO as a biological mediator include cGMP-dependent vasodilation, neural communication, host defense, inflammation, immune suppression, and blood clotting [22]. 

However, overproduction of NO during inflammation can activate nuclear factor kappa B (NF-*κ*B) and induce the expression of proinflammatory mediators, which can promote inflammation by increasing cGMP levels and vascular permeability [22, 23]. NO can be toxic and can cause many diseases, such as cancer and atherosclerosis [24]. NO is produced from L-arginine by inducible NO synthase (iNOS). Overproduction of NO by iNOS can result in cytotoxicity and tissue damage [25]. iNOS is expressed in vascular smooth muscle cells, macrophages, and hepatocytes in response to immune-modulating molecules, such as LPS, interleukin (IL)-1, interferon gamma (IFN-*γ*), tumor growth factor beta (TGF-*β*), and proinflammatory cytokines [22, 26–28]. Thus, inhibition of NO production by the downregulation of iNOS in macrophages is a significant therapeutic strategy in the development of anti-inflammatory agents. In this study we demonstrated that CHA inhibited NO production significantly in LPS-stimulated macrophages (Figure 2), this action appears to involve the inhibition of iNOS gene overexpression (Figure 3(a)). 

On the other hand, COX-2 has an important role in the conversion of arachidonic acid to prostaglandins, especially PGE_2_, a mediator that can result in acute and chronic inflammation [29–31]. The inhibition of COX-2 is clinically relevant because the resulting PG production is thought to be responsible for the antipyretic, anti-inflammatory, and analgesic proprieties of AINEs [32]. COX-2 is certainly a pivotal enzyme in inflammation, and inhibitors of COX-2 are being developed to obtain safer anti-inflammatory drugs. Many studies have demonstrated that compounds that selectively inhibit COX-2 produce less damage to the gastric mucus [1]. Our data also showed the inhibitory effects of CHA on COX-2 mRNA expression in LPS-stimulated macrophages (Figure 3(b)). 

TNF-*α* and IL-6 are small secreted proteins that mediate and regulate immunity and inflammation. Both cytokines are derived mainly from activated macrophages. TNF-*α* has an important role in the immune response and has the ability to prevent infections and to keep inflammation locally circumscribed, but inappropriate or excessive production of TNF-*α* can be harmful [33]. Secreted TNF-*α* further induces cells to release IL-1*β* and IL-6 [34]. IL-6 is one of the most common inflammatory cytokines [35]; it can amplify the inflammatory cascade and cause injury [36, 37]. Moreover, both TNF-*α* and IL-6 are involved in the inflammatory response occurring in the vascular endothelial cells and promote the initiation and evolution of atherosclerosis by causing endothelial cells to express adhesion molecules and induced endothelial dysfunctions [38–40]. Here, we observed that CHA could suppress the production of TNF-*α* and IL-6 from LPS-activated macrophages (Figure 4). Furthermore, CHA may inhibit the mRNA overexpression of both cytokines at the pre-translational level (Figure 5).

The chemical composition of the Lamiaceae family, especially the *Hyptis* genus, is remarkably variable, as they contain compounds such as terpenes, flavonoids, lactones, lignans, phenolic derivatives, and steroids [41]. It is reasonable to assume that the anti-inflammatory activity observed in extract of *H. albida* is due to the synergistic action of many components. Chemical analyses have revealed that the composition of *H. albida* extract consists of flavonoids and triterpenoids including betulinic, ursolic, oleanolic, and acetyl oleanic acids, and these compounds exhibit a variety of interesting medicinal properties [11]. 

Many terpenoids and flavonoids possess anti-inflammatory activities in various animal models of inflammation. Many investigators have proposed cellular mechanisms of action explaining the *in vivo* anti-inflammatory activities of these compounds. The terpenoid ursolic acid is found in many plants and is known to have anti-inflammatory activity [42]. Several authors have reported that ursolic acid suppresses the expression of the proinflammatory enzymes, COX-2 and iNOS [43], and inhibits the production of NO in macrophages [44] and NF-*κ*B activation [45]. Some studies have indicated that some terpenoids have gastroprotective activity that involves reinforcement of defensive factors in the gastric mucosa [46]. On the other hand, flavonoids modulate the activities of arachidonic acid (AA) metabolizing enzymes such as phospholipase A_2_ (PLA_2_) [47, 48], COX-2 [49], and lipoxygenase (LOX) [50–52] and iNOS [53]. Flavone and several amino-substituted flavones were reported to inhibit NO production [54, 55]. The production of some cytokines, such as IL-1, IL-6, and TNF-*α*, was inhibited by flavonoids in LPS-treated human blood monocytes and RAW 264.7 cells [56, 57].

In conclusion, we demonstrate that CHA is a potent inhibitor of inflammation. It inhibited the release of inflammatory mediators from macrophages and suppressed the overexpression of relevant genes. Further studies are underway to isolate the active components from *H. albida* and characterize their mechanism of action.

## Figures and Tables

**Table 1:** List of sequences used for RT-PCR.

Gene	Sequence	Length (bp)
iNOS	Forward: ACCTTGGAGTTCACCCAGT	170
	Reverse: ACCACTCGTACTTGGGATGC	

COX-2	Forward: GCGAGCTAAGAGCTTCAGGA	212
	Reverse: TCATACATTCCCCACGGTTT	

TNF-*α*	Forward: CTGGGACAGTGACCTGGACT	204
	Reverse: GCACCTCAGGGAAGAGTCTG	

IL-6	Forward: AGTTGCCTTCTTGGGACTGA	159
	Reverse: TCCACGATTTCCCAGAGAAC	

18s	Forward: GTAACCCGTTGAACCCCATT	140
	Reverse: CCATCCAATCGGTAGTAGCG	
